# Feasibility of Outpatient Follow-Up After High-Dose Cytarabine Consolidation in Patients With Acute Myeloid Leukemia

**DOI:** 10.7759/cureus.106502

**Published:** 2026-04-06

**Authors:** Md Foys Ullah, Md Gulam Kibria, Kazi Fazlur Rahman, Md Zakaria Hossen, Nahid Sultana, Amin Lutful Kabir, Akhil Ranjan Biswas

**Affiliations:** 1 Department of Hematology, Continental Hospital, Dhaka, BGD; 2 Department of Hematology, Faridpur Medical College and Hospital, Faridpur, BGD; 3 Department of Hematology, Mohanpur Upazila Health Complex, Rajshahi, BGD; 4 Department of Hematology, Dhaka Medical College and Hospital, Dhaka, BGD; 5 Department of Hematology, Bangladesh Medical University, Dhaka, BGD; 6 Department of Hematology, Chittagong Medical College, Dhaka, BGD

**Keywords:** acute myeloid leukemia, consolidation therapy, high-dose cytarabine, neutropenia, outpatient chemotherapy

## Abstract

Background

High-dose cytarabine (HiDAC) consolidation is an essential component of post-remission therapy for acute myeloid leukemia (AML) and is usually delivered in inpatient settings due to anticipated cytopenias, infection risks, and transfusion needs. However, rising healthcare burdens have prompted interest in outpatient administration, but evidence remains limited, particularly in resource-constrained environments. This study aimed to evaluate the feasibility, safety, and clinical outcomes of outpatient follow-up after HiDAC consolidation compared with those of inpatient management.

Methods

This prospective observational study included 50 patients with AML aged 12-60 years who achieved complete remission following induction therapy and subsequently received high-dose cytarabine consolidation at Dhaka Medical College and Hospital from March 1, 2023, to February 29, 2024. Participants were allocated to outpatient (n=25) or inpatient (n=25) follow-up groups based on clinical assessment and physician discretion, including performance status, caregiver availability, and proximity to the hospital. Demographic characteristics, comorbidities, hematologic parameters, adverse events, infection patterns, and healthcare utilization, particularly duration of hospitalization, were compared between the groups. Statistical analyses were performed using independent t-tests and chi-square tests, with statistical significance set at p≤0.05.

Results

Baseline characteristics were comparable across groups. All patients experienced expected cytopenias, with no significant differences in non-hematologic toxicities. Severe neutropenia (absolute neutrophil count (ANC) <0.5×10⁹/L) was less frequent in the outpatient group (7 (28%) vs. 17 (68%); p=0.005), and nadir ANC was significantly higher. Infection rates were low overall, with no infection-related mortality. Outpatient follow-up markedly reduced hospital stay duration (10.76 ± 0.78 vs. 17.2 ± 1.68 days, p<0.001) without increasing complications, readmissions, or documented infections. Patterns of culture-positive infections and hematologic recovery were comparable between settings.

Conclusions

Outpatient follow-up after HiDAC consolidation appears to be a feasible approach and may be safe in carefully selected AML patients based on performance status, caregiver support, and proximity to the hospital, with reduced hospitalization and no observed increase in short-term complications. With structured monitoring and standardized supportive care, outpatient management may help reduce healthcare burden. Larger multicenter studies with longer follow-up are required to validate long-term outcomes and refine patient selection.

## Introduction

Acute myeloid leukemia (AML) is a malignant disorder characterized by the uncontrolled proliferation of clonal myeloid progenitors, leading to heterogeneity in cytogenetic and molecular alterations and variable responses to therapy [1-4]. It may arise de novo or from prior myelodysplastic or therapy‑related damage. The median age at diagnosis is approximately 68 years, and the five‑year overall survival rate remains at 24%-30% [2,3]. Integrated genetic markers divide AML into favorable, intermediate, and adverse risk categories [5,6], and complete remission is achieved in approximately 45% of younger patients and 20% of older adults after standard induction chemotherapy [7]. The 7+3 regimen of seven days of cytarabine plus anthracycline has been the therapeutic backbone for decades, yet many patients relapse and require post‑remission consolidation or salvage therapy [2,4].

Despite incremental advances, overall survival remains poor in patients with relapsed or refractory AML; options are limited to further cycles of cytarabine‑based therapy, and responses often last months [8]. Combinations such as clofarabine plus cytarabine improve remission rates but cause significant infectious complications [9,10]. Targeted agents (e.g., FMS-like tyrosine kinase 3, or FLT3, and isocitrate dehydrogenase, or IDH, inhibitors) have improved outcomes for select patients but are not universally available [11]. For older or unfit patients, lower intensity therapy combining venetoclax with hypomethylating agents is now the standard of care; phase trials have reported complete remission rates of approximately 73% and a median overall survival of 14.7 months, although febrile neutropenia remains frequent [12].

High‑dose cytarabine (HiDAC) is the standard post‑remission consolidation for fit patients with non‑adverse-risk AML. The classic regimen delivers cytarabine at 3 g/m² every 12 h on days 1, 3, and 5 [13]. Randomized trials have shown that, compared with intermediate‑dose schedules, HiDAC improves relapse‑free and overall survival [14] but benefits plateau at doses above 1.5 g/m², particularly in older adults, due to neurotoxicity [15]. Previous studies suggest that intermediate‑dose (1-2 g/m²) cytarabine may offer comparable efficacy with less toxicity [16], yet repetitive HiDAC cycles remain the standard in favorable‑risk AML [17]. Adding anthracyclines, such as idarubicin, did not improve survival or increase infections [18].

HiDAC is usually delivered in patients because clinicians anticipate severe myelosuppression, febrile neutropenia, transfusion needs, and logistical challenges [19]. Infection remains a major concern; plasma proteomic profiling of neutropenic AML patients reveals heterogeneous inflammatory signatures and underscores the difficulty in predicting infection‑related complications [20]. Case reports describe febrile neutropenia, sepsis, and pancreatitis during HiDAC therapy that necessitate early discontinuation and transition to oral therapy [21]. Improved prophylaxis and recognition of nosocomial infection risk, coupled with rising costs, have prompted exploration of outpatient HiDAC treatment [1,22,23]. Outpatient care reduces bed occupancy and may improve patient and caregiver satisfaction [24], but requires careful candidate selection and close monitoring by multidisciplinary teams [24].

Observational studies offer limited data. In a study from the Brazilian Amazon, outpatient HiDAC cycles showed relapse and transfusion rates comparable to those of inpatient therapy; however, 40.74% of the patients required hospitalization during outpatient cycles, and toxicity increased in patients older than 50 years [23]. At a Saudi center, 127 outpatient cycles produced 38 episodes of febrile neutropenia, with no early mortality and more than 62.2% of cycles without complications; investigators concluded that outpatient HiDAC is safe, feasible, and cost‑effective [1]. However, existing reports involve small cohorts and varied protocols, and lack contemporary inpatient controls. This context underscores the need for prospective studies comparing outpatient and inpatient consolidation to guide practice appropriately. Therefore, the primary objective of this study was to compare the duration of hospitalization between outpatient and inpatient HiDAC consolidation. Secondary objectives included comparison of hematologic toxicity, infection rates, adverse events, and healthcare utilization between the two groups.

## Materials and methods

Study design and setting

This prospective observational study was conducted at the Department of Hematology and Bone Marrow Transplant Unit, Dhaka Medical College and Hospital, Dhaka, Bangladesh, between March 1, 2023, and February 29, 2024. This study aimed to assess the feasibility of outpatient follow-up for high-dose cytarabine consolidation chemotherapy in patients with acute myeloid leukemia.

Sample size calculation

The sample size was determined on the basis of the prevalence of AML, which accounts for approximately 1% of all cancers worldwide [25]. Sample size was calculated using the following formula, with a 95% confidence interval and a 5% margin of error:

\[ n = \frac{Z^{2}pq}{d^{2}}. \]

The required sample size was calculated to be approximately 16. However, due to the limited availability of prior comparative data, the sample size estimation was initially based on prevalence. Considering feasibility and patient availability during the study period, a total of 50 patients were ultimately enrolled to allow meaningful comparison between the two treatment settings. The samples were divided into two groups: 25 patients in the outpatient department (OPD) and 25 patients in the inpatient department (IPD).

Sampling technique

A purposive sampling method was used to select participants who met the inclusion criteria. This approach was chosen due to the relatively low incidence of eligible AML patients undergoing HiDAC consolidation within the study period and the need to include all feasible cases in a real-world clinical setting. The inclusion criteria included AML patients aged 12-60 years who achieved complete remission after induction or reinduction therapy. Patients with preexisting infectious diseases or those who had received prior HiDAC treatment outside of the study center were excluded. Informed consent was obtained from all participants, and for those under 18, assent was also obtained. After providing consent, patients were assigned to either the OPD or IPD group based on predefined clinical criteria. Although purposive and non-randomized sampling may introduce selection bias, this study was designed as a feasibility study, and efforts were made to minimize bias by including all consecutive eligible patients during the study period. Furthermore, both adolescent and adult patients were included due to the limited patient pool.

Data collection procedure

Data were collected from each patient via semistructured checklists. The checklist included demographic details, clinical examination findings, and laboratory test results. For baseline data, blood samples were collected for complete blood count (CBC), serum procalcitonin, and blood culture. Follow-up data were collected twice a week during the chemotherapy cycle and for three weeks post-consolidation. Patients in the OPD group were given instructions regarding personal care, and telephone follow-ups were conducted to monitor their progress. In cases of febrile neutropenia, suspected sepsis, or bleeding, patients were advised to return to the hospital for immediate care. Febrile neutropenia was defined as a single oral temperature ≥38.3°C or ≥38.0°C sustained for more than one hour in the presence of neutropenia [26]. Infection was defined based on clinical features and/or microbiological confirmation from blood or urine cultures.

Treatment protocol and monitoring

Patients were assigned to either the OPD or IPD group based on predefined clinical criteria. Patients selected for outpatient follow-up were clinically stable, had no active infection or bleeding, had acceptable performance status, adequate caregiver support, and were able to maintain close follow-up, including telephonic communication and timely hospital access. Patients requiring closer monitoring or with unstable clinical conditions were managed in the inpatient setting. The chemotherapy regimen consisted of high-dose cytarabine administered intravenously at a dose of 3 g/m² every 12 hours on days 1, 3, and 5 of each cycle. All patients received standard premedication with palonosetron and dexamethasone. Supportive care was standardized across both groups and included prophylactic antimicrobials, blood product transfusion as required, and granulocyte colony-stimulating factor (G-CSF) support according to clinical indication. Patients in the OPD group were followed up twice weekly and were additionally monitored through regular telephone follow-up by the hematology team. Patients and caregivers were educated regarding early warning signs such as fever, bleeding, or clinical deterioration, and were instructed to seek immediate hospital care if such symptoms occurred. Patients were instructed to report immediately to the hospital in case of fever, bleeding, or any new symptoms, and were admitted for further evaluation and management when clinically indicated. Blood and urine cultures were obtained when clinically indicated, particularly during febrile episodes or suspected infection, rather than routinely at fixed intervals.

Statistical analysis

The data were analyzed via IBM SPSS Statistics, version 24.0 (IBM Corp., Armonk, USA). Descriptive statistics such as the mean and standard deviation were used for continuous variables, whereas categorical data were analyzed using frequency and percentage distributions. The comparison between the OPD and IPD groups for continuous variables was performed using the two-sample t-test, and for categorical variables, the Pearson chi-square test was applied. A p-value of ≤0.05 was considered statistically significant. The data are presented in tables, figures, and graphs for clarity.

Ethical issues

Ethical approval for this study was obtained from the Ethics Committee of Dhaka Medical College (memo no. ERC-DMC/ECC/2023/27) before data collection began. All study procedures followed the ethical guidelines of both the institutional and the national research bodies, in line with the principles of the 1964 Helsinki Declaration and its subsequent revisions. Written informed consent was obtained from every participant before inclusion in the study.

## Results

Among the 50 patients receiving HiDAC consolidation, 25 were managed in the OPD setting and 25 in the IPD setting. The mean age was 39.36 ± 11.70 years in the OPD group and 36.52 ± 13.72 years in the IPD group. Males comprised 11 (44.0%) of OPD and 9 (36.0%) of IPD patients. Body surface area was similar between groups (1.53 ± 0.12 vs. 1.51 ± 0.12 m²). Most participants in both groups lived more than 5 km from the hospital. Monthly income distribution did not differ significantly, and the majority belonged to 20,000 to 40,000 BDT (Bangladeshi taka) income families (Table 1).

**Table 1:** Baseline demographic and social characteristics of the study participants (n=50) 5 km: distance from the hospital; BDT: Bangladeshi taka; OPD: outpatient department; IPD: inpatient department

Variable	Category	OPD (n=25), n (%)	IPD (n=25), n (%)	p-value
Age (years), mean ± SD		39.36 ± 11.70	36.52 ± 13.72	0.435
Sex	Male	11 (44.0)	9 (36.0)	0.564
	Female	14 (56.0)	16 (64.0)	
Body surface area (m²)		1.53 ± 0.12	1.51 ± 0.12	0.613
Residence	Within 5 km	3 (12.0)	0	0.074
	>5 km	22 (88.0)	25 (100.0)	
Monthly income (BDT)	10,000–20,000	7 (28.0)	8 (32.0)	0.203
	20,000–40,000	15 (60.0)	17 (68.0)	
	>40,000	3 (12.0)	0	

Comorbid illnesses were present in 12 (48.0%) of OPD and 14 (56.0%) of IPD patients (Table 2). Diabetes was noted in 4 (16.0%) and 7 (28.0%), hypertension in 6 (24.0%) and 6 (24.0%), and asthma/COPD in 2 (8.0%) and 1 (4.0%) of OPD and IPD patients, respectively. A total of 13 OPD patients (52.0%) and 11 IPD patients (44.0%) had no comorbidity. A significant difference was observed in the duration of hospitalization, with a mean stay of 10.76 ± 0.78 days in the OPD group compared to 17.2 ± 1.68 days in the IPD group (p<0.001).

**Table 2:** Comorbidities and hospital stay among OPD and IPD patients (n=50) COPD: coronary obstructive pulmonary disease; OPD: outpatient department; IPD: inpatient department p<0.05 was considered statistically significant.

Variable	OPD (n=25), n (%)	IPD (n=25), n (%)	p-value
Diabetes mellitus	4 (16.0)	7 (28.0)	0.761
Hypertension	6 (24.0)	6 (24.0)	
Asthma/COPD	2 (8.0)	1 (4.0)	
No comorbidity	13 (52.0)	11 (44.0)	
Duration of hospital stay (days), mean ± SD	10.76 ± 0.78	17.2 ± 1.68	<0.001

As seen in Table 3, the most frequent adverse events were hematologic. Cytopenia occurred in all patients in both groups. Neutropenia and leukopenia were seen in 25 (100%) of OPD patients and 24 (96.0%) of IPD patients. Thrombocytopenia and anemia were universal in both groups. Non-hematologic toxicities were infrequent. Fatigue was reported in 24 (96.0%) of OPD and 23 (92.0%) of IPD patients. Bone pain occurred in 8 (32.0%) and 7 (28.0%), rash in 2 (8.0%) and 2 (8.0%), conjunctivitis in 9 (0%) and 1 (4.0%), and cerebellar toxicity in 0 (0%) and 1 (4.0%) of OPD and IPD patients, respectively.

**Table 3:** Adverse effects of HiDAC consolidation among OPD and IPD patients HiDAC: high-dose cytarabine; OPD: outpatient department; IPD: inpatient department p<0.05 was considered statistically significant.

Adverse effect	OPD (n=25), n (%)	IPD (n=25), n (%)	p-value
Cerebellar toxicity	0 (0.0)	1 (4.0)	0.312
Cytopenia	25 (100)	25 (100)	—
Neutropenia	25 (100)	24 (96.0)	0.312
Leukopenia	25 (100)	24 (96.0)	0.312
Thrombocytopenia	25 (100)	25 (100)	—
Anemia	25 (100)	25 (100)	—
Conjunctivitis	0 (0.0)	1 (4.0)	0.312
Fatigue	24 (96.0)	23 (92.0)	0.552
Bone pain	8 (32.0)	7 (28.0)	0.758
Rash	2 (8.0)	2 (8.0)	1.000

Severe neutropenia (absolute neutrophil count (ANC) <0.5×10⁹/L) occurred in 7 OPD patients (28.0%) and 17 IPD patients (68.0%) (p=0.005). The mean lowest ANC was 0.65 ± 0.34 ×10⁹/L in the OPD group and 0.38 ± 0.31 ×10⁹/L in the IPD group (p=0.006). The duration of severe neutropenia was similar in both groups. Platelet counts below 25,000/µL were seen in 10 (40.0%) of OPD and 15 (60.0%) of IPD patients. Median day of platelet nadir was day 17 (IQR 15-18) in both groups (Table 4).

**Table 4:** Nadir hematologic values and duration of cytopenia OPD: outpatient department; IPD: inpatient department; ANC: absolute neutrophil count; IQR: interquartile range p<0.05 was considered statistically significant.

Variable	OPD (n=25), n (%)	IPD (n=25), n (%)	p-value
Patients with severe neutropenia (ANC <0.5×10⁹/L)	7 (28.0)	17 (68.0)	0.005
Duration of ANC <0.5 (days), mean ± SD	3.85 ± 1.07	3.94 ± 1.24	0.878
Lowest ANC, mean ± SD (×10⁹/L)	0.65 ± 0.34	0.38 ± 0.31	0.006
Patients with platelets <25,000/µL	10 (40.0)	15 (60.0)	0.157
Duration of platelets <25,000/µL (days)	4.4 ± 0.96	6.33 ± 6.11	0.335
Day of nadir platelet count in the cycle (median IQR)	17 (15–18)	17 (15–18)	1.000

Table 5 shows the pattern of culture-positive infections. Overall, infection rates were low, with most blood and urine cultures being negative in both groups, and no significant difference was observed between OPD and IPD patients.

**Table 5:** Pattern of infection on the basis of culture reports OPD: outpatient department; IPD: inpatient department p<0.05 was considered statistically significant.

Culture	Infection type	OPD (n=25), n (%)	IPD (n=25), n (%)	p-value
Blood culture	Pseudomonas	1 (4.0)	0	0.473
	Methicillin-resistant *Staphylococcus aureus* (MRSA)	0	1 (4.0)	
	*Staphylococcus *spp.	3 (12.0)	1 (4.0)	
	Klebsiella	1 (4.0)	0	
	Moraxella	0	1 (4.0)	
	Negative	20 (80.0)	22 (88.0)	
Urine culture	Escherichia coli	4 (16.0)	2 (8.0)	0.539
	Klebsiella	1 (4.0)	0	
	Proteus	0	1 (4.0)	
	Negative	20 (80.0)	22 (88.0)	

## Discussion

Our cohort included 50 patients who achieved complete remission after induction therapy and proceeded to HiDAC consolidation, with equal distribution between outpatient and inpatient settings. Baseline characteristics, including age, sex, body surface area, and comorbidities, were comparable between the groups, minimizing confounding and allowing a valid comparison of treatment settings. Similar demographic comparability has been reported in a Brazilian cohort study [23].

The present study demonstrated that carefully selected patients can be safely transitioned to outpatient consolidation therapy. This finding supports previous recommendations emphasizing the importance of patient fitness, caregiver availability, and proximity to healthcare facilities when considering outpatient management [24]. However, detailed cytogenetic and molecular risk stratification was not uniformly available in our study, limiting full assessment of biological comparability between OPD and IPD groups, which should be considered when interpreting the findings.

The most notable difference was the significantly shorter duration of hospitalization in the OPD group. This reflects our practice of admitting OPD patients only for chemotherapy administration and early monitoring, followed by discharge once clinically stable, whereas IPD patients remained hospitalized until hematologic recovery. Comparable findings have been reported in the Memorial Sloan Kettering Cancer Center study [19], where OPD patients had substantially fewer hospitalization days per cycle, and in a Saudi Arabian study demonstrating reduced treatment delays, lower bed occupancy, and decreased healthcare costs with outpatient care [1]. Additionally, condensed HiDAC regimens with pegfilgrastim support have been shown to reduce hospital stay without compromising outcomes [17]. These findings collectively support the feasibility and economic advantage of outpatient consolidation therapy.

Hematologic toxicity was universal, with nearly all patients developing neutropenia, thrombocytopenia, and anemia, which is consistent with the known mechanism of cytarabine. Non-hematologic toxicities, including fatigue, bone pain, rash, and conjunctivitis, were infrequent and comparable between groups, aligning with previous reports [19]. Only one case of cerebellar toxicity was observed, consistent with the low incidence reported at doses below 3 g/m² [19]. These findings suggest that outpatient care does not increase treatment-related toxicity.

Severe neutropenia occurred less frequently in the OPD group (28%) compared to the IPD group (68%), although the duration of neutropenia was similar. This difference may be explained by patient selection, as clinically stable individuals were more likely to be managed in the outpatient setting. Standardized supportive care, including G-CSF administration and antimicrobial prophylaxis, may also have contributed. The potential influence of selection bias should therefore be considered. Differences in supportive strategies across studies may also account for variability in outcomes. For example, the Brazilian cohort reported hospitalization in 40.74% of outpatient cycles due to febrile neutropenia or infection [23]. In contrast, no unplanned readmissions were observed among OPD patients in our study, likely reflecting careful patient selection and close monitoring. Previous studies have also shown that pegfilgrastim reduces neutropenia duration and infection risk [17].

Infection rates were low in both groups, with most cultures remaining negative and no infection-related mortality observed. The identified organisms included *Pseudomonas*, methicillin-resistant *Staphylococcus aureus*,* Staphylococcus *spp.,* Klebsiella*,and *Moraxella*, while urinary infections were mainly due to *Escherichia coli*. These findings are consistent with prior studies reporting low infection-related complications in outpatient settings [1,19]. With appropriate prophylaxis and monitoring, outpatient HiDAC does not appear to increase infection risk.

This study has several limitations. First, the single-center design and relatively small sample size (n=50) may limit the generalizability of the findings and reduce the statistical power, particularly for detecting differences in safety outcomes. Second, the non-randomized design and purposive sampling may have introduced selection bias, as patients selected for outpatient management were likely to be clinically more stable and had better caregiver support and access to healthcare facilities. Third, although supportive care was intended to be standardized across both groups, variations in real-world clinical practice, including timing and intensity of monitoring, antimicrobial use, and G-CSF administration, cannot be entirely excluded. Fourth, detailed cytogenetic and molecular risk stratification data according to contemporary classifications were not uniformly available, limiting full assessment of biological comparability between groups. Additionally, important confounding factors such as patient fitness, caregiver availability, and distance from the hospital were not formally controlled, which may have influenced treatment allocation and outcomes. Microbiological confirmation of infections was also limited, with several febrile episodes remaining culture-negative. Furthermore, the study did not assess long-term outcomes such as relapse, survival, or quality of life, which are important for evaluating the overall effectiveness of outpatient management strategies. Finally, inclusion of both adolescent and adult patients without separate subgroup analysis may limit interpretation, as pediatric AML differs in biological behavior and treatment response compared to adult AML. Future studies should include larger, multicenter populations with randomized allocation, standardized supportive care protocols, detailed risk stratification, and long-term follow-up to validate these findings and enhance generalizability.

## Conclusions

This study suggests that outpatient follow-up after high-dose cytarabine consolidation may be safe and feasible for carefully selected AML patients. Hematologic toxicities, infection patterns, and non-hematologic adverse events were comparable between the outpatient and inpatient groups, while outpatient care was associated with reduced hospitalization days without an observed increase in short-term complications. These findings highlight a practical alternative that may ease bed occupancy and improve patient convenience when adequate monitoring systems are in place. However, given the non-randomized design and potential selection bias, these findings should be interpreted with caution. Culture-positive infections and severe neutropenia still occur, underscoring the need for vigilant follow-up. Larger multicenter studies with longer outcome tracking will help refine patient selection and strengthen the evidence supporting outpatient HiDAC management.
